# Effects of resource-dependent cannibalism on population size distribution and individual life history in a case-bearing caddisfly

**DOI:** 10.1371/journal.pone.0191925

**Published:** 2018-02-21

**Authors:** Jun-ichi Okano, Noboru Okuda

**Affiliations:** 1 Center for Ecological Research, Kyoto University, Hirano, Otsu, Shiga, Japan; 2 Research Institute for Humanity and Nature, Motoyama, Kamigamo, Kyoto, Japan; USDA Agricultural Research Service, UNITED STATES

## Abstract

Resource availability often determines the intensity of cannibalism, which has a considerable effect on population size distribution and individual life history. Larvae of the caddisfly *Psilotreta kisoensis* build portable cases from sedimentary sands and often display cannibalism. For this species, the availability of preferable case material is a critical factor that affects larval fitness, and material is locally variable depending on the underlying geology. In this study, we investigated how sand quality as a case material determines cannibalism frequency among larvae and, in turn, how the differential cannibalism frequency affects the body-size distribution and voltinism. Rearing experiments within a cohort revealed that a bimodal size distribution developed regardless of material quality. However, as the preferable material became abundant, the proportion of larger to smaller individuals increased. Consecutive experiments suggested that smaller larvae were more frequently cannibalized by larger ones and excluded from the population when preferable smooth material was abundant. This frequent cannibalism resulted in a bimodal size distribution with a significantly higher proportion of larger compared to smaller individuals. The size-dependent cannibalism was significantly suppressed when the larvae were raised in an environment with a scarcity of the preferable case material. This is probably because larvae cannot enjoy the benefit of rapid growth by cannibalism due to the difficulties in enlarging their case. At low cannibalism the growth of smaller individuals was stunted, and this was probably due to risk of cannibalism by larger individuals. This growth reduction in small individuals led to a bimodal size-distribution but with a lower proportion of larger to smaller individuals compared to at high cannibalism. A field study in two streams showed a similar size distribution of larvae as was found in the rearing experiment. The bimodal ratio has consequences for life history, since a size-bimodal population causes a cohort splitting: only larvae that were fully grown at 1 year had a univoltine life cycle, whereas larvae with a stunted growth continued their larval life for another year (semivoltine). This study suggests that availability of preferable case building material is an important factor that affects cannibalism, which in turn affects larval population size structure and cohort splitting.

## Introduction

Cannibalism is a widespread feeding behavior that occurs across many taxa [1, 2]. Because cannibalism and the resultant population regulation have a profound effect on community and food web dynamics [3–5], understanding the factors that promote and maintain cannibalism in ecosystems is critical across a broad ecological spectrum.

One presumable benefit of cannibalism is the higher nutritional and energetic gain from the consumption of conspecifics rather than alternative food items [6, 7]. However, cannibalism also involves risk, such as acquiring species-specific pathogens, injury, and death through fighting with their victims [8, 9]. Hence, cannibalism occurs only when the benefits outweigh the cost, and its frequency is controlled by various factors, such as nutritional and physiological conditions, resource availability, coefficient of relatedness, and developmental time constraints [6, 10–12].

Animals often exhibit size-dependent cannibalism: larger individuals eat smaller ones because they can more easily subdue or ingest their preys [13, 14]. Size-dependent cannibalism often considerably affects the population structure, such as producing a bimodal size distribution [15, 16]. Several mechanisms can establish bimodality and these mechanisms are not mutually exclusive of each other; cannibals increase their growth rates compared to non-cannibals [17, 18], cannibals selectively prey on individuals in intermediate size classes [19–21], and the growth of individuals in small size classes is stunted owing to risk of cannibalism by those in large size classes [10, 22, 23]. In addition, as size-dependent cannibalism intensifies before the establishment of bimodality, size distribution becomes more skewed toward larger size classes [10, 18].

In insects the number of generations in a year (voltinism) is an important life history characteristics that can vary across environmentally diverse habitats, thereby strongly influencing population growth and individual fitness [24]. Because the developmental rate during the larval stage can vary within a cohort, a bimodal size distribution can cause variation in voltinism, i.e., larger individuals emerge at age 0 (univoltine), whereas smaller individuals continue their larval life for another year (semivoltine) [13]. Therefore, when the population size distribution varies according to cannibalism frequency, voltinism also varies. However, few comprehensive studies have examined how cannibalism frequency is determined and, in turn, how it affects the population size distribution and individual life history. Such knowledge is important for understanding the dynamics of predator–prey systems not only within a species but also among different species [25–27].

Caddisfiy larvae in the family Odontoceridae (Order: Trichoptera) often display cannibalism [27, 28]. The larvae build cylindrical portable cases of sedimentary sands [29]. The availability of preferable case materials is a critical factor that affects the larval fitness. It has been reported that they have a strong preference for smooth-surfaced sand materials (microscale surface texture) [29–31], which improves their respiration efficiency by abdominal undulation, thereby reducing their metabolic costs and mortality [32]. The availability of these preferable smooth materials may also be a limiting factor in larval growth because case enlargement difficulties occur when high quality case material is scarce [30]. This is particularly important when an individual experiences rapid growth by assimilating highly nutritional food as the larvae urgently need to enlarge their cases concurrent with their growth. Given this growth constraint, caddisfly larvae may not have the growth advantage of cannibalism when high quality case materials are scarce. Previous studies also showed that the abundance of smooth sand materials in larval habitat varied locally and was dependent on the mineralogical/petrological constituents of the sediment (e.g., quartz, feldspar, chert, or sand-mudstone) [30, 31]. Therefore, we predicted that the occurrence of cannibalism and its resultant population size distribution and individual life history in odontocerid larvae would be dependent on the availability of optimal case materials in their local habitat.

In this study, using *Psilotreta kisoensis* larvae belonging to the Odontoceridae, we tested three hypotheses that 1) the availability of preferable case materials, in stream habitats affects growth performance and case construction efficiency, 2) case construction efficiency determines the frequency of size-dependent cannibalism, and 3) the resultant occurrence of cannibalism modifies the population size distribution and cause variation in voltinism.

## Materials and methods

The experiment was performed in accordance with all applicable laws and the rules of Kyoto University, and necessary permits were in hand when the research was conducted.

### Study species

The larvae of the case-bearing caddisfly *Psilotreta kisoensis* Iwata are widely distributed in spring-fed and headwaters of mountain streams on Honshu Island, Japan. Larvae enlarge their rigid cylindrical cases by extending the anterior end of the case with sedimentary sands as they grow.

The larvae often display cannibalism and the cannibal usually eats a conspecific by invading the victim’s case [28]. Hence, cannibals do not share their victim with other individuals. This contrasts with other case-bearing caddisfly species that exhibit ‘mob cannibalism’ [33]. Other than conspecifics, we observed that larvae also ate acorns, chironomid larvae and the flesh of dead animals, such as terrestrial arthropods.

This species has variable voltinism which is dependent on their developmental stage in late spring. Larvae can only emerge and reproduce in May, females lay one egg clutch and die. While some larvae reach maturity within a year (univoltine), those that are too immature to emerge during the emergence season continue larval life for another year until the following May (semivoltine).

### Study sites and habitat quality

We surveyed the larval size distributions of wild populations in the permanent small streams of Mt. Aoba (38°15′N, 140°49′E, Site 1) and Mt. Maya, Japan (34°43′N, 135°11′E, Site 2). We also conducted laboratory experiments using the Site 2 population whose individuals were reared in one of three types of sedimentary sands collected from these two sites and the Kakita River (35°6′N, 138°54′E, Site 3). In Site 2, the range of physical and chemical characteristics were water temperature (4–23 °C), water discharge (0.0062–0.012 m^3^/s), total nitrogen (0.29–0.54mg/l), and total phosphorus (0.002–0.010 mg/l) [34]. The stream was not influenced by drying or seasonal ice.

**Fig 1:**
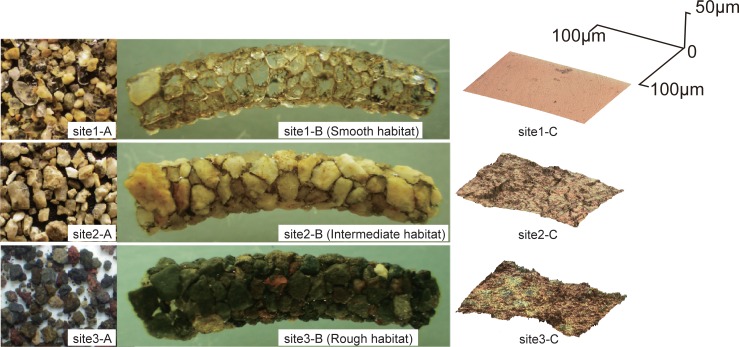
Images of sand materials. Sand materials in the sediment (A), larval case (B), and a 3D image of the microscale surface texture of representative sand materials from the larval case (C) at each location. The 3D images (C) were referenced from Okano et al. (2011).

Definition of surface roughness (i.e., smoothness) and availability of smooth-surfaced materials in three habitats have been described in detail by Okano et al. [30, 31]. In short, surface roughness (*Ra*) is defined by the arithmetic average of roughness profile absolute values within a 40 × 40 μm area of material surface, quantified using confocal laser scanning microscopy (Fig 1C). Site 1 is abundant in smooth-surfaced quartz materials (i.e., lower *Ra*) that originated from volcanic ash, whereas Site 3 comprises scoria sands and is scarce in smooth materials (Fig 1) [30]. Site 2 comprises granitic sands and is intermediate between Sites 1 and 3 in relation to the abundance of smooth materials [30]. Although larvae chose smoother materials from the surrounding sediment to construct cases in all three habitats, the end product had a rougher surface in habitats where smooth materials were scarce (Fig 1; site 3-C). The availability of smooth materials can be a limiting factor for case construction, but smooth materials are not considered to be a competitive resource as they are continually replenished by rock erosion.

### Laboratory experiments

#### Habitat manipulation

We manipulated the habitat quality for case building larvae in relation to the availability of preferable case materials (i.e., smooth materials) to examine its effects on population characteristics, the occurrence of cannibalism, and the efficiency of case construction. Sedimentary sands were collected from Sites 1, 2, and 3, each of which varied in their abundance of smooth materials (hereafter referred to as case-material “smooth,” “intermediate,” and “rough” habitats, respectively). After the desiccation of sedimentary sands in laboratory, the sand particles were sorted into four size classes using sieves (<0.25 mm, 0.25–0.5 mm, 0.5–1.0 mm, and 1.0–1.4 mm) for each of the three sites. We then mixed these four size classes in the proportion of 1:4:4:4, respectively, by weight within each site to standardize their granularity.

#### Experiment 1: Population characteristics

We examined how the availability of smooth materials affects population characteristics, such as growth, survival, the resultant size distribution, and voltinism, by allotting larvae to one of three habitat types under the condition of rearing in isolation or in a group. We could not perform the rearing experiment on the Site 1 population because these larvae had a low growth rate in the rough habitat. This was probably due to their extremely strong preference for smooth materials [30]. We also conducted rearing trials on other populations (*P*. *kisoensis* from Site 5 and *Perissoneura paradoxa* [from the same Odontoceridae family] from Sites 4 and 7; the site numbers were referenced from Okano et al. [30]), and they showed population characteristics similar to those observed in the Site 2 population. Thus, here we focus only on the results from the Site 2 population.

At the beginning of June, we collected 32 egg clutches from Site 2 and hatched them in containers (L × W × D: 28 × 20 × 8 cm) containing sedimentary sands from Site 2. We only used the larvae that appeared within the first 3 days of hatching to control their initial body size.

At the beginning of July, 1080 immature larvae were randomly assigned to 12 containers (L × W × D: 13 × 13 × 5 cm) with 135 mL of sedimentary sands derived from one of three habitat types under the group-rearing condition (90 individuals × three habitat types × four replicates; two replicates in 2012, one replicate in 2014, and one replicate in 2015). The experimental period was 12 months during which larvae were fed fish flakes (Tetra Fin; Tetra Co., Melle, Germany). The nutritional contents of the fish flakes were 49% of proteins and 12% of fats, which is almost similar to the natural food of chironomids (26–56% of proteins and 10–14% of fats [35, 36]) but higher than acorns (*Quercus crispula* and *Quercus acutissima*, 5–6% of protein and 2–3% of fats [37]). The food ration was 6 mg every day until September and then 18 mg every 3 days. Compared to the oligotrophic condition in their natural habitats, this food ration was considered sufficient because some fish flakes were not consumed before the next feeding. Surplus food was removed before each feeding to keep the water clean. We supplied aged tap water for all containers and renewed half of the water volume every 2 weeks. To simulate the natural environment, the sedimentary sands were replaced every month to maintain a constant supply of smooth materials in each habitat. Container locations were reassigned within an aquarium every 6 days to eliminate any spatial effects.

During the experimental period, we counted the number of individuals in each container once in 2–3 months to examine the survival rate. We also measured the aperture diameter (AD, mm) at the anterior end of their cases under a binocular microscope (×30 magnification) to estimate their body size. AD was converted to larval body weight (mg dry weight, DW) on the basis of the conversion equation derived by Okano et al. [27]. Based on the equation, there was no significant difference in the initial larval body weight among the three habitat types (ANOVA, year 2012: F_2, 117_ = 0.29, p = 0.75; year 2014: F_2, 57_ = 0.10, p = 0.90; Year 2015: F_2, 57_ = 0.033, p = 0.97; 20 subsamples from each container).

To eliminate any intraspecific interaction effects on individual growth and mortality under the group-rearing condition, each of the 168 larvae were reared in isolation in a small container (L × W × D: 4 × 4 × 4 cm) with one of the three habitat types (56 individuals × three habitat types; N = 28 in 2012 and N = 28 in 2014 for each habitat type). There was no significant difference in the initial larval body weight among the three habitat types (ANOVA, year 2012: F_2, 81_ = 0.14, p = 0.87; year 2014: F_2, 81_ = 0.029, p = 0.97). The isolated-rearing condition was the same as the group-rearing condition, except that the larvae were individually fed in isolated containers. Although the food ration was 0.2 mg every 3 days at the beginning of the experiment, we gradually increased it to 0.4 mg toward the end of the experiment. This was the same as food amount in the group rearing with the assumption that larval survival was 50% in the group rearing later in the experiment. Similarly, we also increased sedimentary sands in a container from 1.5 mL to 3 mL. We regarded the difference in mortality between the group- and isolated-rearing conditions as larval death caused by cannibalism.

#### Experiment 2: Cannibalism frequency

We examined how the availability of smooth materials affects the frequency of cannibalism occurrence by conducting a follow-up experiment to explain any differences in the mortality among the three habitat types as detailed in Experiment 1. In Experiment 2, we used larvae hatched from 22 egg clutches collected from Site 2 in June and allocated them to one of three habitat types (N = 127 for case-material smooth habitat, N = 158 for intermediate habitat, and N = 158 for rough habitat; because survival was lower in the rough and intermediate habitats, we allocated more individuals to those habitats to obtain enough specimens). Each was reared in isolation in a small container (L × W × D: 4 × 4 × 4 cm). Because larval growth was different among individuals and habitat types, the rearing period varied 3–5 months until the start of the experiment to set same condition of body size distribution. The feeding regime was the same as isolated-rearing in Experiment 1.

Between September and November, when the immature larvae reached around 0.74 mg body weight based on the conversion equation [27], we picked 15 individuals from the small containers in one of the three habitats and transferred them to a middle-sized container (L × W × D: 7 × 7 × 4.5 cm) without sedimentary sands to rear in a group (15 individuals × three habitat types × four replicates; N = 3 in 2014, and N = 1 in 2015). The initial larval size distribution was normal (unimodal) and not significantly different among habitat types (ANOVA, Year 2014: F_2, 132_ = 0.21, p = 0.81; Year 2015: F_2, 42_ = 0.069, p = 0.93; abundant: 0.74 ± 0.098 mg DW, intermediate: 0.74 ± 0.091 mg DW, scarce: 0.74 ± 0.099 mg DW). The larvae were starved in isolation for 5 days prior to being transferred to induce their foraging activity, although they were fed fish flakes *ad libitum* during the experiment under the group-rearing condition.

Over the 30 h experiment (20°C, 6L:12D:12L), some cases were observed to be empty, suggesting that their owners were cannibalized by others in the same container. Indeed, we observed some cannibalism occurrences. After 30h, we measured AD for the empty and occupied cases to estimate their individual body size. Because we did not supply sedimentary sands, the larvae could not enlarge their cases. After AD was measured, the remaining larvae were returned to small containers where they were raised in isolation, allowing them to enlarge their cases with sedimentary sands for 5 days. Fish flakes were withheld for 5 days to prevent larval death due to hunger. Subsequently, AD was measured again to estimate the growth rate of the remaining larvae during these 5 days. With the assumption that cannibals had a higher growth rate, we distinguished cannibal from non-cannibal survivors using growth rate data (see results) and retroactively determined the body weight of cannibals at the end of the experiment. Apart from this, the body weight of victims could be estimated from AD of empty cases at the end of the experiment.

#### Experiment 3: Case construction efficiency

We examined how the availability of smooth materials affects the efficiency of case construction by conducting a follow-up experiment. In February 2015, 36 fully grown larvae were collected from Site 2 and assigned to one of three habitat types (N = 12 for each habitat type). There was no significant difference in the larval size by habitat type (ANOVA, F_2, 33_ = 0.078, p = 0.93; smooth: 1.7 ± 0.24 mg DW, intermediate: 1.7 ± 0.30 mg DW, rough: 1.7 ± 0.25 mg DW). We removed the anterior part of the case (ca. one-third of its length) to induce case reconstruction. Each individual was introduced into an isolated plastic container (L × W × D: 4 × 4 ×4 cm) with 3 mL of sedimentary sands derived from one of three habitat types, and the containers were placed in a large aquarium tank filled with aged tap water (20°C, 12L:12D). We marked the anterior of unremoved parts with a pencil to discriminate between the unremoved and newly constructed parts of the case. At 30 h after case removal, we measured the length of the case reconstructed by the larvae under a binocular microscope to evaluate the efficiency of case construction.

### Field survey

We conducted a field survey to compare the size distribution and voltinism of wild populations between Sites 1 and 2. Using a quadrat frame (L × W × D: 25 × 25 × 4 cm), sedimentary sands were collected in triplicate from the stream sediment in sluggish flow areas (pools and edges of the stream) at each site where larvae typically inhabit. In the laboratory, we sorted the larvae, estimated the larval density, and measured their AD alive to estimate body size, based on the equation [27].

### Statistical procedure

For all statistical analyses, we used R v. 3.1.0 software (http://www.r-project.org/). For descriptive purposes, means ± SD are given.

#### Growth rate (Experiment 1)

To examine the growth advantage of using smooth case materials, we compared the temporal change in the larval body size among the three habitat types under the isolated-rearing condition. We used chi-squared test with post-hoc Tukey’s method (“multcomp” R package) after fitting a generalized linear mixed model (GLMM; “lme4” R package) with gamma errors. Year (2012 and 2014) was incorporated as the random intercept to consider the unknown effects of using larvae from different clutches by year and habitat type as the random slope.

#### Size-distribution and voltinism (Experiment 1)

To statistically analyze the size distribution modality in each of the experimental and the wild populations, we used normal mixture modeling (“mclust” R package), which can detect discrete peaks in the size distribution, assuming that it forms a Gaussian distribution, according to Bayesian information criterion [38]. Because the normal mixture modeling generally detected a bimodal size distribution (i.e., size splitting into larger and smaller larvae) in group-reared populations from November to April, we calculated the numerical proportion of larger to smaller larvae (L:S) as an index of skewness in the size distribution. The L:S ratios and voltinism (proportion of univoltine to semivoltine) in group-reared populations were compared among the three habitat types using chi-squared test with post-hoc Tukey’s method after fitting a GLMM with a binomial distribution. We incorporated year (2012, 2014 and 2015) as the random intercept and the habitat type as a random slope.

#### Survival rate (Experiment 1)

In each of the group- and isolation-reared populations, the survival curves were compared among the three habitat types using chi-squared test with post-hoc Tukey’s method after fitting a Cox proportional hazard model (CPHM; “survival” R package) to incorporate each container and year as the random intercept into the model.

#### Cannibalism frequency (Experiment 2)

To compare the frequency of cannibalism in each container among the three habitats, we used chi-squared test with post-hoc Tukey’s method after fitting the GLMM with a binomial distribution and incorporating year (2014 and 2015) as a random intercept into the model (three sediment habitats × four replicates). To estimate which individuals predated conspecifics in the containers (i.e., cannibals), a Gaussian normal mixture modeling was applied to the enlargement rate of AD during the 5 days after the cannibalism experiment, with an assumption that the nutritional benefit of cannibalism increased the growth rate of the cannibals. We expected that a bimodal distribution would be detected if cannibals experienced rapid growth and enlarged their cases, compared with other survivors. After the estimation, to confirm whether the larvae displayed size-dependent cannibalism, we compared the body sizes of cannibals and victims at the end of the 30-h experiment using Student’s t-test. Data were available only for the case-material smooth habitat because cannibalism rarely occurred in the other two habitats.

#### Case construction efficiency (Experiment 3)

We compared the length of the reconstructed case among the three habitat types using ANOVA with Tukey’s post-hoc test.

## Results

### Growth rate

**Fig 2:**
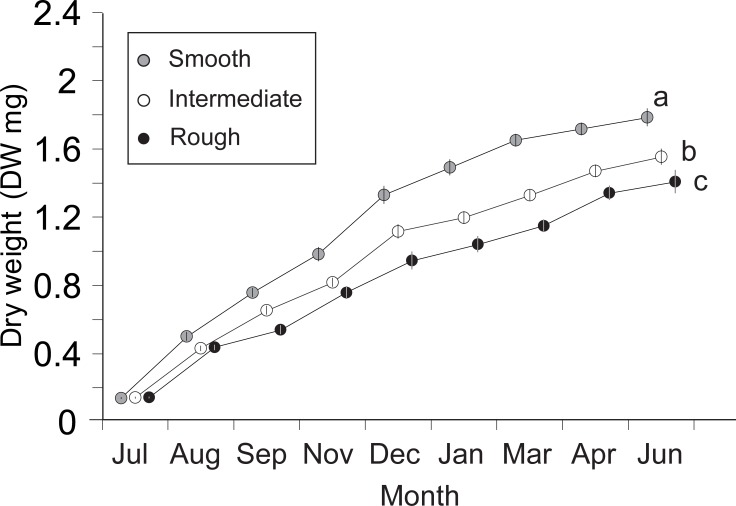
Growth of larvae reared in isolation in three habitat types (mean ± SE). Different letters indicate significant differences (Tukey’s test, p < 0.001).

In Experiment 1, where each larva was reared in isolation, there was significant difference in the larval growth among the three habitat types (chi-squared test, χ^2^ = 52, df = 2, p < 0.001). The larvae grew significantly larger in the case-material smooth habitat than in the intermediate (Fig 2; Tukey’s test, z = 4.3, p < 0.001) and rough habitats (z = 6.3, p < 0.001), and grew larger in the intermediate habitat than in rough habitat (z = 2.7, p = 0.018).

### Size distribution and voltinism

**Fig 3:**
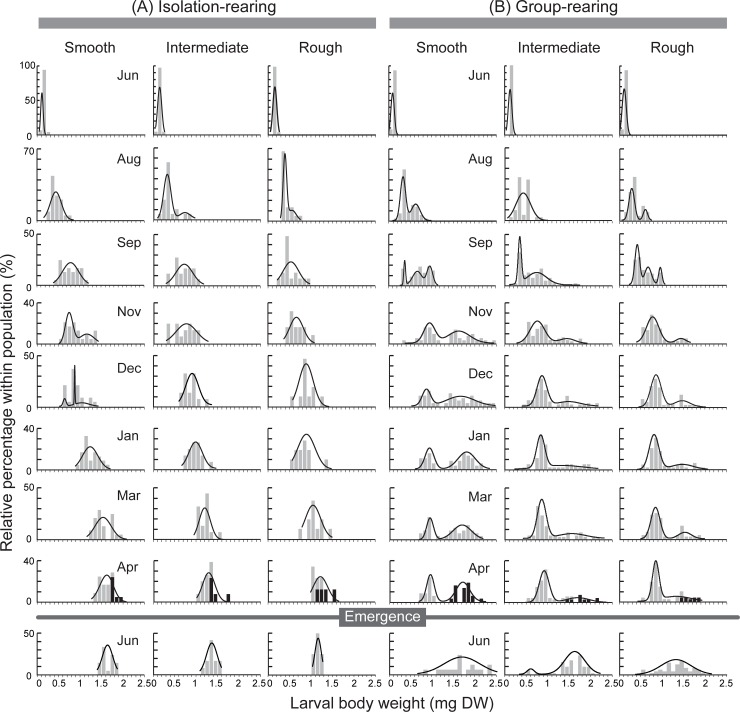
**Size distribution of larvae reared in three habitat types under (A) isolated-condition and (B) group-condition**. Data for 2012 is showing representatively. Black bars in April indicate emerging larvae. Lines indicate best-fitted multimodalities using Gaussian normal mixture modeling.

When each larva was reared in isolation, the size distributions did not show a marked bimodality (Fig 3A, S1 Table). By contrast, when larvae were reared in a group, the size distributions shifted from unimodal with a narrow peak just after the hatching to bimodal with a broader size range (Fig 3B). After November, the bimodal size-distributions stagnated. After the emergence of large-sized larvae, the small-sized larvae grew up and continued their larval life for another year. During the growing season in the group-rearing condition, the size distributions were highly skewed toward the larger size classes in the case-material smooth habitat. The L:S ratio was significantly higher in the smooth habitat than in the intermediate and rough habitats (Tukey’s test, smooth vs. intermediate: z = 13, p < 0.001; smooth vs. rough: z = 15, p < 0.001; intermediate vs. rough: z = 2.3, p = 0.050; after all over chi-squared test, χ^2^ = 290, df = 2, p < 0.001).

There was significant difference in voltinism among the three habitats as reflected by the L:S ratio in the group-rearing condition, as larger mature larvae could emerge after one year (black bar on April in Fig 3B, S1 Table). The proportion of univoltine to semivoltine was significantly higher in the smooth habitat (1.2 ± 0.25) than in the intermediate (0.19 ± 0.051) and rough habitats (0.083 ± 0.043; Tukey’s test, smooth vs. intermediate: z = 5.9, p < 0.001; smooth vs. rough: z = 7.1, p < 0.001; intermediate vs. rough: z = 2.0, p = 0.11; after chi-squared test, χ^2^ = 69, df = 2, p < 0.001).

### Survival rate and cannibalism

**Fig 4:**
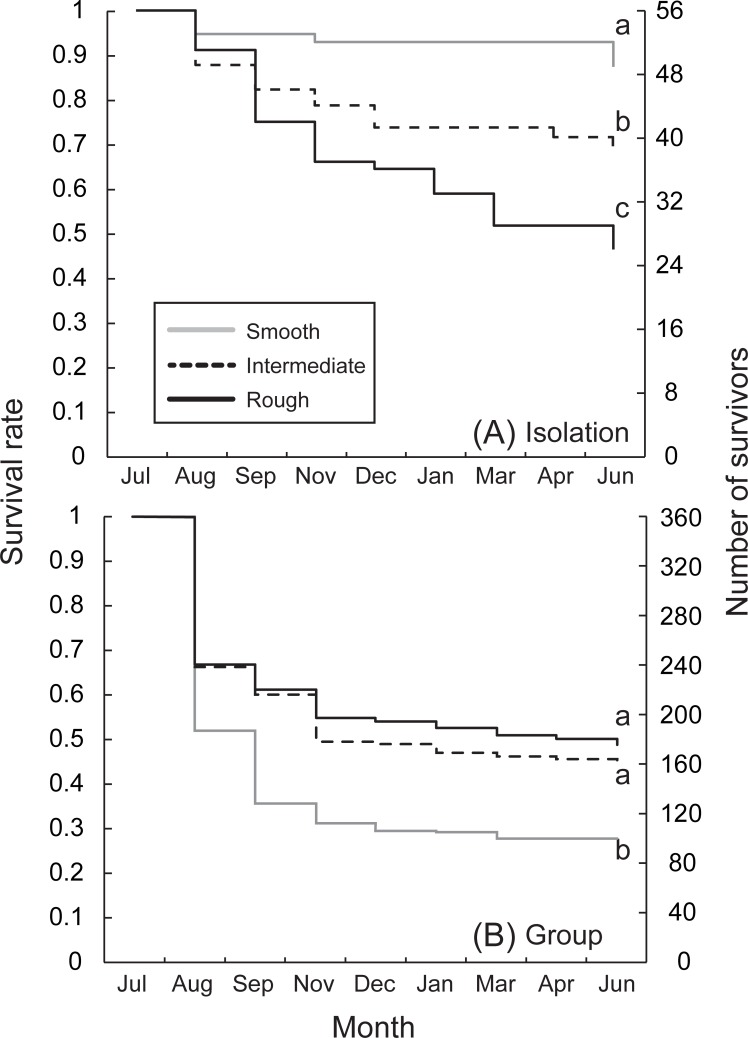
**Survival of larvae incubated in three habitat types in (A) isolation- and (B) group-rearing conditions.** Distinct letters indicate significant differences among conditions (Tukey’s test, p < 0.05).

When there were no intraspecific interactions under the isolated condition in Experiment 1, the survival rate was significantly higher in the case-material smooth habitat than in the intermediate and rough habitats (Fig 4A; Tukey’s test, smooth vs. intermediate: z = 2.5, p = 0.029; smooth vs. rough: z = 4.3, p < 0.001), and higher in the intermediate habitat than rough habitat (intermediate vs. rough: z = 2.4, p = 0.044; after chi-squared test, χ^2^ = 26, df = 2, p < 0.001). Conversely, when they were reared in a group, the effect of habitat quality on the survival rate was reversed, i.e., the survival rate was significantly lower in the smooth habitat than in the other two habitats (Fig 4B; Tukey’s test, smooth vs. intermediate: z = 13, p < 0.001; smooth vs. rough: z = 15, p < 0.001; intermediate vs. rough: z = 2.3, p = 0.05; after chi-squared test, χ^2^ = 38, df = 2, p < 0.001). However, after November when a bimodal distribution was established, mortality gradually declined in all habitats, indicating that cannibalism mostly occurred before a bimodal distribution was established.

**Fig 5:**
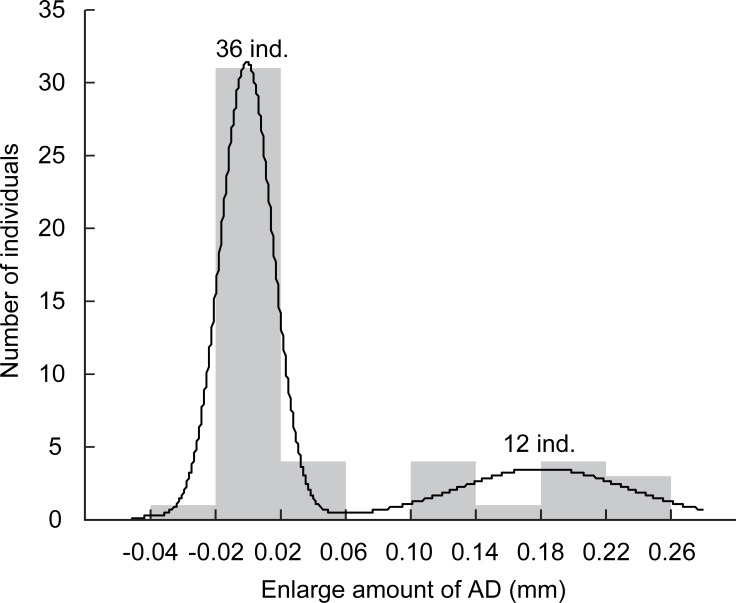
Distribution of the amount of cases with enlarged aperture diameter (AD) of survivors 5 days after the cannibal experiment. Line indicates best-fitted multimodalities using Gaussian normal mixture modeling.

**Fig 6:**
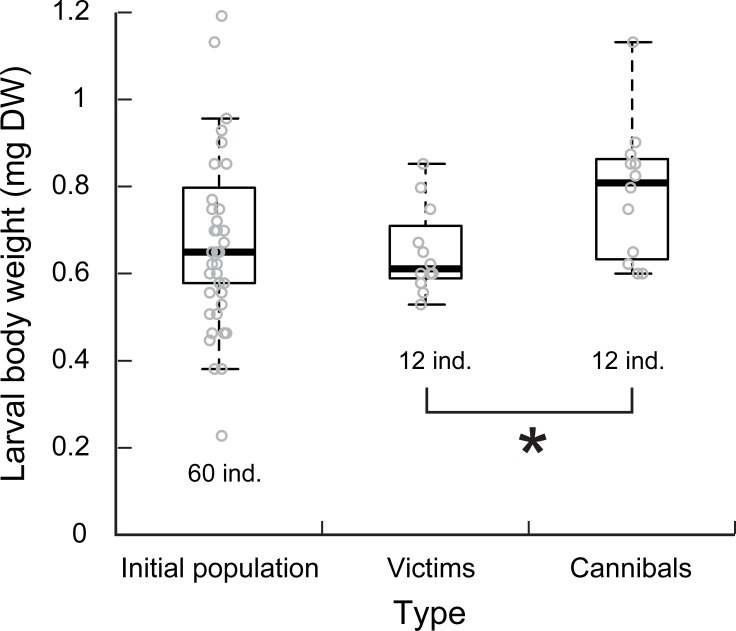
Body weight before the cannibal experiment under the case-material smooth habitat. Box plots show median, first and third quartile and 95% confidence interval of median. Circles indicate actual measurement values. We identified larvae that grew significantly larger as cannibals after the experiment. *indicates the significant difference in weight between the victims and cannibals, as obtained by t-test.

In Experiment 2 where larvae were reared in a group, cannibalism occurred more frequently in the case-material smooth habitat than in the other two habitats (S1A Fig; number of victims in smooth habitat = 3.0 ± 0.82, intermediate = 0.5 ± 0.58, rough = 0.25 ± 0.50; Tukey’s test, smooth vs. intermediate: z = 2.5, p = 0.028; smooth vs. rough: z = 2.6, p = 0.027; intermediate vs. rough: z = 0.57, p = 0.82; after chi-squared test, χ^2^ = 16, df = 2, p < 0.001). Five days after the end of the group-rearing, a marked bimodal distribution in the enlargement rate of case sizes was observed (Fig 5), indicating that some survivors had a higher growth rate than others after the experiment. The number of individuals with such a higher growth rate (4, 3, 3, and 2 individuals for each trial) was just in accordance with that of the empty cases (4, 3, 3, and 2 individuals for each trial). The circumstance of having same number of individuals in both indicates that the former were cannibals and the latter were victims, with an assumption that the nutritional benefit of cannibalism increased their growth rate. The body size estimated from AD just after the 30 h experiment was significantly larger for cannibals (0.85 ± 0.14 mg DW) than for victims (Fig 6; 0.75 ± 0.097 mg DW, *t*-test, *t* = 1.7, df = 22, p = 0.023). Considering that the larvae could not enlarge their case under the experimental condition without sand materials, this result suggests that the initial body size was larger for the cannibals than for the victims in the experimental population (i.e. size-dependent cannibalism).

### Case construction efficiency

Experiment 3 revealed that larvae could extend their cases more efficiently in smooth habitat than in other two habitats (S1B Fig; length of repaired case in smooth habitat = 3.4 ± 1.1 mm, intermediate = 2.1 ± 1.4 mm, rough = 1.8 ± 1.3 mm; Tukey’s test, smooth vs. intermediate: p = 0.012, smooth vs. rough: p = 0.043, intermediate vs. rough: p = 0.86; after ANOVA, F_2, 33_ = 5.3, p = 0.010).

### Wild populations

**Fig 7:**
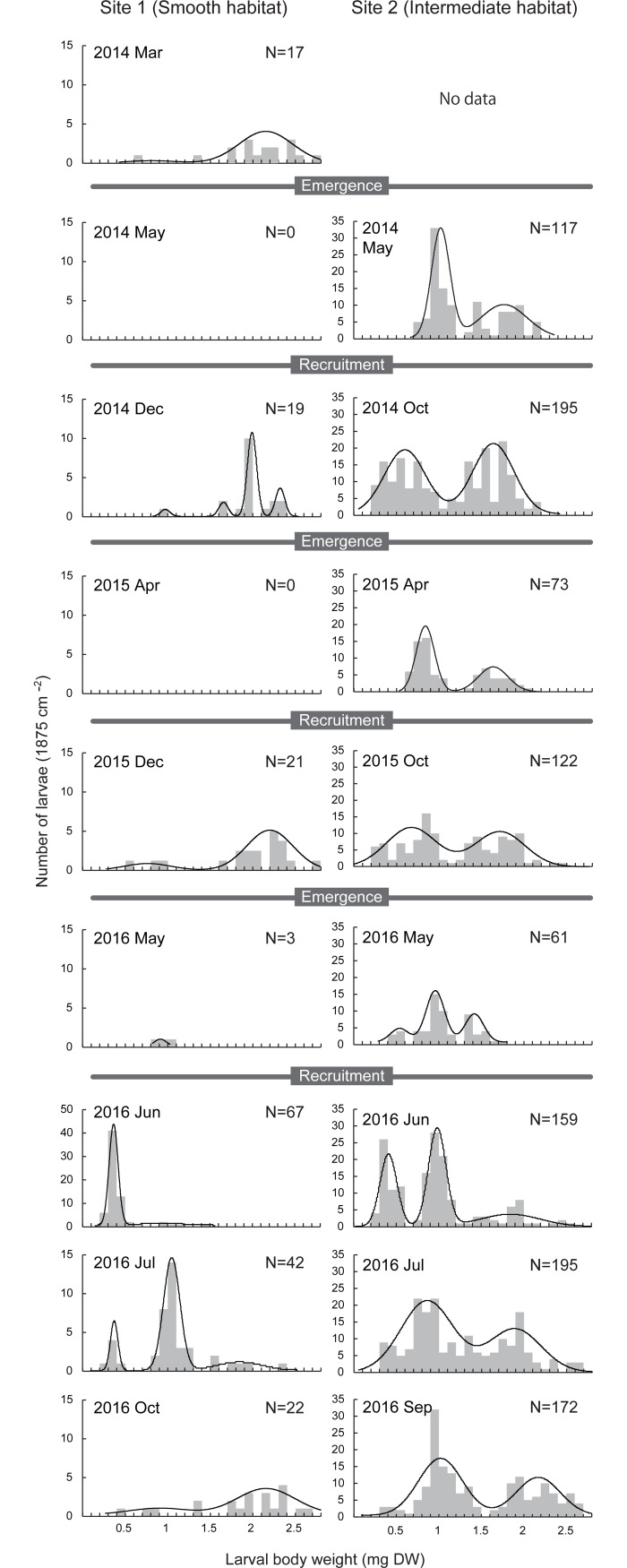
Size distribution of wild larvae. Left panels are in a case-material smooth habitat (Site 1) and right panels are in intermediate habitat (Site 2). Lines indicate best-fitted multimodalities using Gaussian normal mixture modeling.

At Site 1 (case-material smooth habitat), the size distribution was skewed toward smaller size classes just after hatching, which then shifted toward larger size classes during the developmental season (Fig 7, S2 Table). In this population, few larvae were found in the emergence season, suggesting that most of the larvae reached a mature size and emerged at age 1 (i.e., univoltine). By contrast, at Site 2 (case-material intermediate habitat), the size distribution skewness was moderate but a clear bimodality occurred throughout the year. Even after the emergence season, a considerable number of larvae remained, suggesting that some individuals postponed their emergence to the next season (i.e., semivoltine).

## Discussion

### Development and maintenance of bimodal size distribution

We found that a bimodal size distribution appeared in group rearing, in which the L:S ratio was higher when size-dependent cannibalism frequently occurred. In addition, the cannibals enlarged their cases after assimilating conspecifics. Thus, the likely developmental process of a bimodal size distribution is that a slight difference in body size determines cannibals and non-cannibals [17, 22, 39], and cannibals increase their growth rates by assimilating high nutrition conspecifics compared to non-cannibals [6, 18, 40]. However, the observed cannibalism and its resulting bimodality were dependent on material quality, being more frequent when smooth materials were abundant.

After the size bimodality was established, cannibalism rarely occurred and the size-distribution stagnated. However, the smaller larvae could grow after the larger larvae had emerged. These processes indicate that the growth of smaller larvae was stunted in the presence of larger cannibals. Stunted growth in a size-structured population is often explained by food competition; larger cannibals are not subject to food competition, whereas smaller non-cannibals suffer from food competition [41–44]. However, in our rearing conditions, we provided adequate food for the larvae. An alternative possible factor for their stunted growth was the risk of cannibalism by the larger larvae. It is known that the foraging activities of non-cannibals are often reduced by the presence of cannibals [45–48]. Another possibility is that smaller larvae delay their own growth to escape the size window of cannibalism—i.e., the preferred intermediate sized conspecifics. In addition to an upper size limit of victims, there is some evidence that cannibals also have lower prey size limit [21]. Although *P*. *kisoensis* larvae eat a conspecific by invading its case, they cannot invade the case of too small ones [28]. The results from our experiment show a similar tendency that middle-sized individuals were typically victims (Fig 6). In most trials where cannibalism occurred, the mean body weight difference between cannibals and victims was within the 27% size range (7%, 8%, 19%, and 10% for smooth; 27% and 12% for intermediate; 16% for a rough habitat). Therefore, once larvae lag behind in their growth, subsequent stunted growth may be a strategy to reduce the risk of cannibalism.

### Differential cannibalism frequency

We further found that the availability of preferable case materials affected the cannibal frequency, which, in turn, had significant effects on the skewness of size distribution and individual voltinism. For the odontocerid caddisfly, smooth materials are an essential case material resource [30, 31] as roughened materials lower the respiration rate, probably because the friction between the surface of the roughened case wall and the larval body depresses abdominal undulation for respiration [49]. Okano et al. [32] estimated a 20%–26% respiration rate decrease in larvae in a roughened-walled case using artificial sands. Therefore, depressed larval growth and increased mortality in isolated-rearing under a scarcity of smooth materials may be due to friction stress.

A scarcity of smooth materials also decreased cannibalism frequency because of a lower efficiency of case enlargement. As a result, the higher mortality arising from roughened case materials was compensated for by the decrease in the frequency of cannibalism (“compensated mortality” [50]). Cannibals rapidly enlarged their cases after consuming conspecifics. However, in an environment where smooth materials were scarce, it would take more time to search for preferable smooth materials to extend their cases. Any delay in case completion may result in an improper fit and a decrease in the total benefit of the case [30]. An improperly fitting case may increase predation risk by exposing soft tissue and/or decrease respiratory efficiency by preventing undulatory movements [30]. In addition, we sometimes observed dead larvae with a folded abdomen in their case. Larvae often turn their body within their case, but the improper-sized case may cause larval death if they become jammed. If the case fit is not perfect the larvae have to enlarge their cases using the least preferred roughened materials, which could decrease their fitness accordingly. Hence, cannibalism frequency might be determined by the balance of growth rate and efficiency of case enlargement. Cannibalism frequency can also decrease as a result of lower predatory aggressiveness arising from the bad conditions [51] such as low quality case materials. However, in our further study, cannibalism was revived after completion of case enlargement when larvae were fully grown (DOI: 10.6084/m9.figshare.5808279.v1; DOI: 10.6084/m9.figshare.5816643.v1). Therefore, we suggest that the cannibalism frequency in our study system was caused by an inefficient case enlargement and not by bad conditions.

Suppressed cannibalism increased larval survival and decreased the L:S ratio as a result of less energy extracted from smaller larvae by larger larvae. This lower L:S ratio further led to a reduction in the ratio of univoltine to semivoltine individuals. In many insect species, voltinism is flexible based on the environmental conditions controlling larval development [52, 53]. Stoks et al. [52] reported that the predation risk could accelerate the generation time of damselfly larvae by increasing the larval growth rate. One of the possible reasons is that larger damselfly larvae swim faster and have a higher probability of survival following an attack [26]. In contrast, predator interference from the redear sunfish *Lepomis microlophus* decreased the growth rate and delayed the generation time of dragonfly larvae [54]. In our study, smaller larvae showed a stunted growth due to pressure from larger ones, which produced cohort splitting; larger larvae had a univoltine life history, whereas smaller larvae switched to a semivoltine life history. Similar cohort splitting has been reported in the larvae of some dragonfly species [55–57].

The size-distribution, density and voltinism of two wild populations were consistent with our experimental populations. Since we evaluated frequency of cannibalism only for larvae from Site 2, we cannot definitely exclude the possibility that the different population characteristics between the two sites was due to any genetic variations but not plasticity of cannibalism behavior. Nonetheless, our further study shows that the larval density was significantly lower in habitats with more abundant smooth particles among eight odontocerid populations (i.e., positive relationship with the roughness of the particles in the natural case; DOI: 10.6084/m9.figshare.5807508.v1; DOI: 10.6084/m9.figshare.5816649.v1; see Okano et al. [27, 30] for population details). This suggests there may be frequent cannibalism in habitats with abundant smooth particles. However, the skewness of size distributions in wild populations was more prominent than in experimental populations. The L:S ratios before the emergence season were 1.1–2.0 (smooth habitat) and 0.2–0.4 (intermediate habitat) in our experimental populations compared with 3.4–16 (Site 1, smooth habitat) and 0.5–1.4 (Site 2, intermediate habitat) in the wild populations. This overproportion in the size modality may be a result of more intense cannibalism due to oligotrophic conditions. In addition, second year generations would be included in the larger size classes in the wild population, which we did not consider in our experimental populations. Further studies will reveal cohort cycling and population dynamics in wild populations in more detail.

## Supporting information

S1 Fig (A) Number of cannibalism victims among larvae raised in case-material smooth, intermediate, and rough habitats. (B) Case length repaired in three types of sediment during 30 h. Boxes indicate mean values with standard deviation. Circles indicate actual measurement values. Different letters indicate significant differences (Tukey’s test, p < 0.05).
(EPS)

S1 Table Results of the statistical analysis of modalities of reared population using the mclust model judged with Bayesian information criterion. Individual numbers classified into each mode (body weight: mean ± variance) are shown. Data from two replicates of each condition in group-rearing is pooled in 2012. L/S indicates the proportion of mode2 (larger individuals) to mode1 (smaller individuals) in a given population after November when bimodal size distributions were commonly established (mean value of two replicates).
(XLSX)

S2 Table Results of the statistical analysis of modalities of a wild population using the mclust model judged with Bayesian information criterion. Individual numbers classified into each mode (body weight: mean ± variance) are shown.
(XLSX)
