# Correction: Effects of resource-dependent cannibalism on population size distribution and individual life history in a case-bearing caddisfly

**DOI:** 10.1371/journal.pone.0197014

**Published:** 2018-05-30

**Authors:** Jun-ichi Okano, Noboru Okuda

The first author’s name appears incorrectly in the citation. The correct citation is: Okano J, Okuda N (2018) Effects of resource-dependent cannibalism on population size distribution and individual life history in a case-bearing caddisfly. PLoS ONE 13(2): e0191925. https://doi.org/10.1371/journal.pone.0191925
